# Protocol for the economic evaluation of the InTENSE program for rehabilitation of chronic upper limb spasticity

**DOI:** 10.1186/s12913-020-05333-z

**Published:** 2020-05-27

**Authors:** Rachel Milte, Julie Ratcliffe, Louise Ada, Coralie English, Maria Crotty, Natasha A. Lannin

**Affiliations:** 1 Caring Futures Institute, Flinders University, Adelaide, South Australia 5001 Australia; 2 Discipline of Physiotherapy, The University of Sydney, Sydney, Australia; 3 School of Health Sciences and Priority Research Centre for Stroke and Brain Injury, University of Newcastle, Newcastle, Australia; 4 College of Medicine and Public Health, Flinders University, Adelaide, Australia; 5 Rehabilitation Services, Flinders Medical Centre, Adelaide, Australia; 6 Department of Neurosciences, Central Clinical School, Monash University, Melbourne, Australia; 7 Alfred Health, Melbourne, Australia; 8 John Walsh Centre for Rehabilitation Research, The University of Sydney, Sydney, Australia

**Keywords:** Rehabilitation, Botulinum toxin, Therapy, Motor training, Costs, Cost effectiveness analysis, Quality of life

## Abstract

**Background:**

Assessment of the costs of care associated with chronic upper-limb spasticity following stroke in Australia and the potential benefits of adding intensive upper limb rehabilitation to botulinum toxin-A are key objectives of the InTENSE randomised controlled trial.

**Methods:**

Recruitment for the trial has been completed. A total of 139 participants from 6 stroke units across 3 Australian states are participating in the trial. A cost utility analysis will be undertaken to compare resource use and costs over 12 months with health-related quality of life outcomes associated with the intervention relative to a usual care comparator. A cost effectiveness analysis with the main clinical measure of outcome, Goal Attainment Scaling, will also be undertaken. The primary outcome measure for the cost utility analysis will be the incremental cost effectiveness ratio (ICER) generated from the incremental cost of the intervention as compared to the incremental benefit, as measured in quality adjusted life years (QALYs) gained. The utility scores generated from the EQ-5D three level instrument (EQ-5D-3 L) measured at baseline, 3 months and 12 months will be utilised to calculate the incremental Quality Adjusted Life Year (QALY) gains for the intervention relative to usual care using area-under the curve methods.

**Discussion:**

The results of the economic evaluation will provide evidence of the total costs of care for patients with chronic upper limb spasticity following stroke. It will also provide evidence for the cost-effectiveness of adding evidence-based movement therapy to botulinum toxin-A as a treatment, providing important information for health system decision makers tasked with the planning and provision of services.

## Background

People with spasticity following stroke have significantly higher care costs (particularly direct healthcare costs, and aged care costs) and lower quality of life than those survivors without spasticity [1–3]. Therefore, identifying effective therapies to reduce upper-limb spasticity and improve function are an important target for research.

International clinical guidelines support the use of botulinum toxin-A in conjunction with active rehabilitation as the preferred treatment [4]. However, the optimum rehabilitation strategy remains undetermined. There are a lack of adequately powered randomised controlled trials evaluating the effect of botulinum toxin-A injections alone, compared to the injection plus active rehabilitation. However, consideration of the costs of providing care for these patients and ultimately consideration of the cost effectiveness of new therapies (namely, whether they are a worthwhile spend of the constrained resources of the healthcare budget as compared to other potential therapies) is another important factor [5].

There have been few studies of the economic impact of upper-limb spasticity following stroke. Lundström et al. [2] evaluated the healthcare costs for the year following stroke in those with and without spasticity in Sweden, and identified that direct health care costs were four times higher in those with spasticity compared to those without, predominantly due to increased costs of hospital care and post hospital community care (i.e. home help services, residential care etc). However, this study only included hospitalised patients and was based on only 25 participants with spasticity. More recently in the UK, Raluy-Callado [3] evaluated costs of care in over 2900 post-stroke spasticity patients and found that those with spasticity following stroke had double the healthcare costs of those without spasticity with increased hospital care contributing to increased costs in this group, but were not able to include information on home and community care in their estimate. In addition, the potential economic impacts of spasticity following stroke are broad ranging, with loss of workforce productivity among patients and their caregivers which persisit after the event [6]. However, the potential cost-effectiveness of therapies is under-researched, with no economic evaluations to date evaluating the impact of evidence-based movement training combined with botulinum toxin-A injections [1, 7, 8]. Rychlik et al. 2016 evaluated the impact for the health care costs and quality of life of botulinum toxin-A treatment vs usual care without botulinum toxin-A. The study showed a significant improvement in the physical and mental health status of participants over the follow up period. Increased healthcare costs were evident for the participants who received the treatment, but despite higher incremental costs (driven by higher pharmaceutical and nursing home care costs) the study authors concluded the intervention was very likely to be considered cost effective due to the large gains in quality of life attributed to the intervention group compared to usual care. However a key limitation of this study was that it was not randomised and the results may have been influenced by confounding factors in the treatment and usual care groups [1]. Conversely, the BoTULS trial evaluated the clinical and cost effectiveness of treating upper-limb spasticity with botulinum toxin-A plus physical therapy vs physical therapy alone over a 4 week intervention period. The study authors concluded that the intervention had a low probability of cost-effectiveness compared to usual care using the UK reference care willingness to pay threshold of £20,000 for an additional QALY gained [9].

In addition, there is an absence of studies from an Australian perspective. Makino et al. 2018 [8] have published the only Australian based study which evaluated the cost-effectiveness of extending botulinum toxin-A therapy beyond the four treatments currently supported by the Pharmaceutical Benefits Scheme. This study was undertaken from the health-care payer perspective, and therefore included direct healthcare costs in the Markov-state transition model that was developed. It was found that extending the number of treatments beyond four was likely to be considered cost effective. However, the study authors didn’t include costs or benefits from rehabilitation or physical therapy in addition to the botulinum toxin-A in their analysis.

The cost of botulinum toxin-A injections is significant, calculated as $1673 Australian Dollars per treatment cycle and patients may receive multiple cycles of treatment [4, 8]. The InTENSE trial [10] aims to determine the clinical and cost effectiveness of including evidence-based movement training with botulinum toxin-A injections. Therefore, interventions to improve the long-term effect of botulinum toxin-A injections in this group could assist in improving quality of life of patients and reducing their healthcare and broader community care costs. Here we describe in detail the protocol for the economic evaluation to occur alongside the evaluation of clinical effect for the InTENSE trial.

## Methods

The clinical protocol for the trial has been described in detail previously [10]. In summary, this Phase III Clinical Trial aims to determine clinical effectiveness of undertaking evidence-based movement training following a botulinum toxin-A injection in adults with neurological spasticity. The study is a national, multicentre randomized clinical trial with concealed allocation, blinded assessment and intention-to-treat analysis. The sites for the study can be found listed on the entry on the Australian and New Zealand Clinical Trial Registry for the Phase III Clinical Trial (ANZCTR12615000616572, date registered 12/06/2015). Recruitment for the study has been completed, with a total of 139 participants from six stroke units across three Australian states participating in the trial. The rational for the sample size for the study has been described in the main clinical protocol paper [10] and was calculated to detect a difference of seven points in the main clinical outcome of the Goal Attainment Scale T-score between the groups with 80% power with a two-tailed significance level of 0.05.

The main objective of the economic evaluation is to undertake a cost-utility analysis of the InTENSE evidence-based movement training with botulinum toxin-A injections as compared to botulinum toxin injections alone. A secondary aim is to undertake a cost-effectiveness analysis of the same using Goal Attainment Scaling (GAS) as the measure of benefit. A third aim, is to determine the costs associated with care for people with spasticity following stroke in a 12 month period in an Australian context.

### Measurement and valuation of costs

A summary of the sources of cost data and valuations are shown in Table 1. The total costs associated with delivering the intervention, including therapist intervention and travel time, consumables, and overheads are collected using a specially designed intervention tracking sheet. Costs of care collected include medical care costs (for example hospitalisation, doctors visits, allied health professional attendances) and non-medical care costs (for example home assistance). Resource use will be collected via a number of methods, using routine administrative datasets where possible. Data on tertiary health service utilisation, including in-patient hospital stays, emergency department presentations, and outpatient presentations are collected from participants via monthly diary over the 12 month follow up period. Use of out-of-pocket or privately funded health and community care services are also collected via this method. The majority of primary health care (such as visits to General Practitioners, and access to prescription pharmaceuticals) in Australia are delivered by the federal government through the Department of Human Services. Data on the use of the Medicare Benefits Schedule (MBS) and the Pharmaceutical Benefits Scheme (PBS) [11] will be collected for consenting participants. Costs for relevant healthcare resources will be sourced from publicly available published data where available and as recommended by guidelines for conducting economic evaluations, including the National Hospital Cost Data Collection, MBS and PBS [12–15]. All costs will be updated to a standard reference year for analysis. Discounting will not be necessary as the follow up period for the study will be 1 year.

**Table 1:** Measurement and valuation of costs

Cost	Specification	Source of Data	Source of Unit Cost
Intervention	Therapist intervention, and travel time, consumables and equipment, overheads	Intervention tracking sheet	Varies. Derived from hospital finance department data.
Public health care costs	General practitioner or Specialist visits, laboratory tests, radiological investigations	Medicare BenefitsSchedule	Medicare Benefits Schedule Fee
Pharmaceuticals	Prescription medications	Pharmaceutical Benefits Scheme	Pharmaceutical Benefits Scheme Dispensed Price for Maximum Quantity
Hospitalisation	Private and publicly funded inpatient admissions	Participant Monthly Diary	National Hospital Cost Data Collection
Emergency Department Presentations	Private and publicly funded emergency department presentations and admissions	Participant Monthly Diary	National Hospital Cost Data Collection
Outpatient visits	Private visits to Allied Health	Participant Monthly Diary	Department of Veteran Affairs, Aged Care Funding Instrument and other relevant datasets as required
Community care services	Domestic assistance, aged care services	Participant Monthly Diary	Department of Veteran Affairs, Aged Care Funding Instrument, and other relevant datasets as required

### Cost-analysis

The total costs of care for participants in the intervention and control groups over the 12 month follow up period will be calculated. This will be aggregated into the health care costs (including costs of hospitalisation, emergency department presentations, doctors attendances, community nursing care attendances, and visits to allied health professionals). In addition, non-medical care costs will be collected and documented, including domestic assistance at home. Costs associated with providing the intervention will be recorded and reported separately. The proportion of participants who utilize the various health services and non-medical care services will be presented, along with the mean and median number of utilizations over the 12 month period. Both the mean and median total costs over the 12 month follow up period for health services and non-medical care will be presented, given cost data is generally of a skewed distribution [16, 17]. The costs of health services and non-medical care will be combined to provide a total cost of care for people with upper-limb spasticity following stroke.

### Measurement and valuation of benefit

The benefit of the intervention will primarily be measured by comparing the quality adjusted life years (QALY) gained in the intervention group as compared to the control group over the 12 month follow up period. Change over time in Health-related quality of life (HrQOL) will be measured using the EQ-5D-3 L [18]. This five-item instrument measures HrQOL across five dimensions (mobility, self-care, usual activities, pain/discomfort, and anxiety/depression) and includes a global health rating using the visual analogue scale (EQ-VAS). It has been widely shown to have excellent validity, including in people with stroke [19]. Participants will complete the EQ-5D-3 L to describe their current HrQOL at baseline (prior to randomisation), at 3 months and again at 12 months. EQ-5D-3 L responses will be converted into a utility score anchored on a scale between 0 (indicating a health state equivalent to death) and 1 (indicating near-perfect health) using the available preference-weights generated from an Australia general population sample [20]. These utility scores will be converted to quality-adjusted life year (QALY) gains for each individual participant over the 12 month follow-up period in by combining data regarding the EQ-5D-3 L health-states of participants with information about the time spent in those health states, using area under the curve methods [21]. In addition, as a secondary analysis, we will use the primary clinical outcome for the trial, the Goal Attainment Scale (GAS) [22]. GAS is a validated measure of achievement of rehabilitation specific goals and measured at the three and 12 month trial timepoints. Goals for their rehabilitation are identified by the participants themselves, as they relate to activity and participation in meaningful tasks. Participants score themselves for current and expected levels of performance (ranging from − 2 to + 2) and t-scores calculated as described by Kiresuk et al. [23].

### Statistical analysis

Descriptive statistics will be presented along with simple statistical tests of difference. Given cost data is generally of a skewed distribution, statistics for normally and non-normally distributed data will be used as appropriate [16, 17]. To adjust for any differences in baseline covariates in the sample (for example in symptom severity), regression analysis of the costs and QALY will be applied. Standard linear regression approaches will be explored, as well as generalized linear regression models which can account for skewed distribution and heteroscedasticity in the data while maintaining the original scale of the data [24, 25]. Where significant levels of missing data occur (5% or greater of the observations), approaches to account for missingness will be undertaken in the analysis [26]. Multiple imputation will be undertaken to account for data missing at random or missing completely at random [17].

### Cost-effectiveness analysis

The primary economic analysis will be carried out on an intention-to-treat basis within a Cost Utility Analysis framework. Additional analyses using a clinical sub-group (those who had mobility through the arm at the beginning of the study assessed as those who could move one or more blocks on the Box and Block Test [27]) will also be investigated. The mean differences in costs and outcomes for the two groups will be presented [16]. The analysis will present the additional resources used (i.e. costs) for an improvement in the outcomes (i.e. QALYs) associated with a new health intervention are compared to usual care (i.e the control group). The result will then be presented as an incremental cost effectiveness ratio (ICER) which is a measure of the additional cost for each unit of improvement in the outcome, and the main outcome will be the incremental costs divided by the incremental QALY gain. The fundamental calculation for the ICER comparing the intervention and control groups will be *ICER* = (*Ca* − *Cb*) ÷ (*Ea* − *Eb*), where *Ca* is the cost of the intervention, *Cb* is the cost of the control, *Ea* is the effectiveness of the intervention and *Eb* is the effectiveness of the control measured in QALYs) [5].

There are multiple potential scenarios that may occur when the costs and benefits of the intervention are combined into an ICER which can be represented by the four quadrants on a cost-effectiveness plane [28]. The intervention may be more effective than the control and be less costly (scenario A), the intervention may be more effective than the control and be more costly (scenario B), the intervention may be less effective than the control (or no different in effect) and be more costly (scenario C), or the intervention may be less effective than the control and more costly (scenario D). Therefore, in the case that the intervention is found to be more effective than the control, the analysis of the costs associated and calculation of the ICER will occur to determine whether the intervention falls into scenario A or B. By comparison, if the intervention is found to be less effective (or no different in effect) to the control, the analysis of costs associated and calculation of the ICER will occur to determine whether the intervention falls into scenario C or D.

For economic evaluations alongside clinical trials, it is recommended to undertake sensitivity analyses to determine the reliability of the results from the analysis, to give an estimate of the level of uncertainty in the findings to take into account in policy decision making [26]. Sensitivity analysis will be undertaken using non-parametric bootstrapping to provide the confidence ellipse, which reflects the uncertainty in the estimate of the ICER. The ellipse provides a region on the cost-effectiveness plane that should contain x% (e.g. 95%) of the uncertainty [29].

Uncertainty regarding the cost-effectiveness of the intervention will be summarized using a cost-effectiveness acceptability curve (CEAC). This curve provides a graphical presentation of the probability that the intervention is cost-effective (has an ICER below the cost-effectiveness threshold) compared with the alternative intervention, given the data, for a range of values for the cost-effectiveness threshold. The CEAC therefore will represent the likelihood of the intervention being cost-effective at a range of ceiling willingness-to-pay thresholds for an additional QALY [29].

## Discussion

This study represents one of the few analyses conducted internationally of the costs of care associated with chronic upper-limb spasticity post stroke, and the first cost-utility study to assess the economic benefit of evidence-based movement training in conjunction with botulinum toxin-A for treatment of upper-limb spasticity post stroke. There is increasing demand for information on not only the clinical effectiveness of healthcare interventions but also their cost-effectiveness, to provide policy makers with critical information regarding the best value for money spend of finite budgets. As such, randomised controlled trials (RCT) of the effectiveness of interventions can provide good opportunities to conduct an economic evaluation alongside the trial, provided the appropriate steps are taken from the outset to ensure that the design of the RCT is fit for this purpose [26]. The economic evaluation for the InTENSE has been considered from the inception of the study, thus allowing an appropriate design and measurement and valuation of costs and benefits to be undertaken within the clinical trial. Our protocol has been planned using available national and international guidelines for conducting economic evaluations, promoting greater transparency in the methods undertaken and increasing the rigor and validity of the findings [11, 26].

This study also represents a unique opportunity to evaluate the economic impact of upper-limb spasticity in a sample of participants post-stroke. There have been few studies of the costs of care for those with upper-limb spasticity following stroke, but those that have been undertaken have identified two to four fold increases in health care expenditures relative to those without spasticity, largely driven by increased hospital and community-based care costs [2, 3]. Accurate information on the costs of care for those with spasticty following stroke is essential for decision-makers in planning for continuing care services available and to understand the potential value-for-money of strategies to support these individuals. In conclusion, this study will provide essential policy-relevant information for decision makers regarding the value of evidence-based movement training for those undergoing an expensive treatment for this life-changing condition.

## Supplementary information

**Additional file 1.**

## Data Availability

The datasets generated and/or analysed during the current study are not publicly available due to restrictions on the publication of the data placed by the Human Research Ethics Committees and third party providers of data. Data may be available from the corresponding author on reasonable request and subject to the approval of any relevant Human Research Ethics Committees and third party providers of data.

## Abbreviations

CEAC: Cost-effectiveness acceptability curve
GAS: Goal Attainment Scale
HrQOL: Health-related quality of life
ICER: Incremental cost effectiveness ratio
MBS: Medicare Benefits Schedule
PBS: Pharmaceutical Benefits Scheme
QALY: Quality adjusted life years
RCT: Randomised controlled trial
