# Supplementary material for: Protocol for the economic evaluation of the InTENSE program for rehabilitation of chronic upper limb spasticity
Source: BMC Health Serv Res. 2020 May 27;20:478. doi: 10.1186/s12913-020-05333-z (PMC7254740; doi:10.1186/s12913-020-05333-z)
Supplement: Supplementary file 1 — Additional file 1. [file 12913_2020_5333_MOESM1_ESM.docx]

**Additional File 1**

**Table 2:** All items from the World Health Organization Trial Registration Data Set.

| **Data category** | **Information** |
| --- | --- |
| Primary registry and trial identifying number | Australia and New Zealand Clinical Trials Registry  ANZCTR12615000616572 |
| Date of registration in primary registry | 12/06/2015 |
| Secondary identifying numbers | N/A |
| Source(s) of monetary or material support | National Health and Medical Research Council |
| Primary sponsor | La Trobe University, La Trobe University, Bundoora, VIC, 3086 |
| Secondary sponsor(s) | N/A |
| Contact for public queries | *A/Prof Natasha Lannin A/Prof Natasha Lannin, Alfred Health, N.Lannin@latrobe.edu.au* |
| Contact for scientific queries | *A/Prof Natasha Lannin A/Prof Natasha Lannin, Alfred Health, N.Lannin@latrobe.edu.au* |
| Public title | Intensive rehabilitation after botulinum toxin-A injections in stroke. |
| Scientific title | *Effect of adding intensive upper limb rehabilitation to botulinum toxin-A on upper limb activity after stroke: the InTENSE trial.* |
| Countries of recruitment | Australia |
| Health condition(s) or problem(s) studied | Stroke |
| Intervention(s) | An evidence-based therapy program is provided for 12 weeks immediately following botulinum toxin-A injection. The program commences with:  (1) Up to 3 serial casts applied to place the wrist in maximum extension for 2 weeks. Cast is applied the first working day following botulinum toxin-A injection; followed by  (2) 10 weeks of movement training, aimed at decreasing weakness (electrical stimulation and progressive resistance exercises) and improving movement (task-specific practice). Participants are encouraged to practice for 1 hour per day, 7 days a week during the 10 weeks (ie, approximately 70 hours in total inclusive of clinic based sessions). Participants are supported by a mix of clinic-based sessions, home visits, and phone calls. Clinic-based sessions will be conducted by a trained physical or occupational therapist (duration 1 hour, frequency decreasing (3 x per week initially, but decreasing to 1 x week by week 7)). Participants will receive a booklet outlining their movement training and a training log to record the number of minutes of practice daily.  Comparator: Written instructions plus follow-up telephone call post-clinic, as per usual care in Australia. Participants will receive a booklet containing 7 stretches, and 8 arm and hand exercises and a training log to record the number of minutes of practice daily. |
| Key inclusion and exclusion criteria | Key inclusion criteria:  - Scheduled to receive a botulinum toxin-A injection to a muscle(s) that crosses the wrist  - Agreed to receive BoNT-A injections as part of their usual care  - Date of stroke three or more months prior.  - Not currently receiving upper limb rehabilitation  - Absence of significant cognitive impairment (as assessed by a score of less than five adjusted errors on the Short Portable Mental Status Questionnaire).  >18 years  Both males and females eligible  Key exclusion criteria:  - Unable to attend clinic at least 1/wk.  - Other significant upper limb impairment eg. Fracture or frozen shoulder within 6 months, severe arthritis, amputation.  - Presence of any and all contraindications to botulinum toxin-A injections.  - Botulinum toxin-A injections and/or serial casts in the past 6 months. |
| Study type | Interventional Allocation: randomized Intervention model: parallel assignment Masking: Blinded (masking used) Primary purpose: treatment Phase III |
| Date of first enrolment | 3/07/2015 |
| Target sample size | 136 |
| Recruitment status | Recruitment completed. |
| Primary outcome(s) | Upper limb activity will be measured using the Box and Block Test at Baseline (pre randomisation), 12 weeks and 12 months.  Individualised achievement of goals will be measured using the Goal Attainment Scale at 12 weeks and 12 months. |
| Key secondary outcomes | Spasticity will be measured using the Tardieu scale  Passive wrist extension will be measured using torque-controlled goniometry.  Health related quality of life will be measured using the EQ-5D  Grip strength will be measured as a maximum voluntary contraction using dynamometry  Pain will be measured using a 10-cm visual analogue scale  Burden of care will be measured using the Carer Burden Scale  All these outcomes will be measured at Baseline (pre-randomisation), 12 weeks and 12 months. |
